# SIRT1 in Senescence: Mitochondria and Immune Crosstalk

**DOI:** 10.3390/biology15181573

**Published:** 2026-09-08

**Authors:** Jirapat Namkaew, Pornparn Kongpracha, Tanakamol Mahawan, Thiranut Jaroonwitchawan

**Affiliations:** 1 Futuristic Science Research Center, School of Science, Walailak University, Thasala, Nakhon Si Thammarat 80160, Thailand; jirapat.na@mail.wu.ac.th; 2 Research Excellence Center for Innovation and Health Products (RECIHP), School of Allied Health Sciences, Walailak University, Thasala, Nakhon Si Thammarat 80160, Thailand; 3 School of Science, Walailak University, Thasala, Nakhon Si Thammarat 80160, Thailand; 4 Quantitative Biosciences Institute (QBI), University of California San Francisco, San Francisco, CA 94143, USA; jo.kongpracha@ucsf.edu; 5 Department of Cellular and Molecular Pharmacology, University of California San Francisco, San Francisco, CA 94143, USA; 6 Data Science and Biotechnology Institute, Gladstone Institutes, San Francisco, CA 94143, USA; 7 Akkhraratchakumari Veterinary College, Walailak University, Thasala, Nakhon Si Thammarat 80160, Thailand; tanakamol.ma@wu.ac.th; 8 One Health Research Centre, Walailak University, Thasala, Nakhon Si Thammarat 80160, Thailand

**Keywords:** SIRT1, cellular senescence, mitochondria, mitophagy, SASP, aging

## Abstract

Cellular senescence is one of the hallmarks of aging. These growth-arrested cells actively secrete inflammatory mediators that reshape the tissue microenvironment and fuel age-related pathology. Sirtuin 1 (SIRT1) is an NAD^+^-dependent deacetylase that regulates senescence largely through its control over mitochondrial integrity and inflammatory signaling. SIRT1 levels and activity fall with age, and this decline directly promotes senescence. SIRT1 maintains mitochondrial function through three interconnected pathways: PGC-1α-driven mitochondria biogenesis, FOXO-dependent antioxidant defense, and mitophagic clearance of damaged organelles. When SIRT1 activity is in an unsteady state, mitochondria become unhealthy. This leads to excessive ROS generation and the leakage of mitochondrial DNA (mtDNA) into the cytosol, which activates the innate immune pathway, consequently resulting in the production of inflammatory cytokines that further inhibit SIRT1. This self-amplifying loop drives cells to irreversible senescence. In this study, we integrate the current understanding of the SIRT1–mitochondria–immune axis within the framework of senescence by examining the biological roles of SIRT1 and the mechanisms that lead to its reduction with aging, while also exploring the interrelated mitochondrial pathways and inflammatory signaling. Furthermore, we assess possible therapeutic strategies targeting this axis and highlight essential questions that necessitate additional research.

## Simple Summary

Aging is the key risk factor for a variety of diseases, such as heart disease, diabetes, and dementia. One of the hallmarks of aging is cellular senescence, a condition where cells stop dividing but remain metabolically active, thus becoming detrimental to the surrounding tissues. This review focuses on a protein known as SIRT1, a molecular guardian of our cells. SIRT1 plays a critical role in maintaining mitochondria health and in suppressing inflammation. Levels of SIRT1 decrease with age, which leads to mitochondrial dysfunction and the release of mitochondrial DNA into the cytoplasm, triggering inflammation. This inflammation further reduces SIRT1 levels, creating a cycle that accelerates cellular aging. This review discusses the most recent and relevant SIRT1, mitochondrial and inflammatory-related findings and highlights prominent strategies, such as increasing SIRT1 activity, to promote healthier and longer lives.

## 1. Introduction

Aging is the primary risk factor for most chronic diseases, including neurodegenerative disorders, cardiovascular diseases, metabolic syndromes, and several types of cancer [1,2]. At the cellular level, the process of aging is characterized by the build-up of senescent cells. Senescent cells have ceased to divide yet continue to be metabolically active, releasing inflammatory factors via the senescence-associated secretory phenotype (SASP) [3]. Although this SASP contributes to wound healing and tumor suppression in the short term, its prolonged presence fosters a tissue-damaging inflammatory environment that hastens the aging process [4,5,6].

Understanding how senescence is initiated and maintained is a major focus for aging research. SIRT1 has emerged as a key player. SIRT1, the mammalian homolog of the yeast longevity assurance gene Sir2, senses the energy state of the cell through its reliance on NAD^+^, whose levels decline with age [7,8]. SIRT1 is involved in regulating metabolism, inflammation and oxidative stress responses, and the cell cycle. SIRT1 functions by deacetylating a variety of targets, including histones, transcription factors, and DNA repair proteins. Due to its functions, SIRT1 involves multiple pathways related to senescence. A shared characteristic among various cell types is the notable reduction in both the binding activity and expression levels of SIRT1 during instances of replicative and stress-induced senescence. This reduction is associated with the onset of senescence [9,10,11]. What causes the decline of SIRT1? Several factors contribute to this phenomenon. These include transcriptional repression, microRNA-mediated suppression of translation, post-translational modifications promoting degradation, and the age-related decrease in NAD^+^, which all contribute to SIRT1 decline. Being interconnected, these various mechanisms ensure that SIRT1 activity diminishes with age from many different angles.

Why does losing SIRT1 cause such profound consequences for senescence? The answer is linked, in particular, to its regulation of mitochondrial function. Mitochondria play a crucial role in senescence. They release reactive oxygen species, their own DNA triggers inflammatory pathways upon release into the cytosol, and their impairment both contributes to and is a consequence of senescence [12]. SIRT1 preserves mitochondrial integrity along multiple converging pathways. It stimulates PGC-1α to enhance mitochondrial biogenesis [13], enhances FOXO-mediated antioxidant defenses [14], and facilitates the mitophagy process [15]. SIRT1 controls a mitochondrial quality control system to ensure that mitochondria stay functional and free from inflammation. When this quality control system fails, the consequences are widespread. The reduction of SIRT1 compromises mitochondrial quality control. This leads to the buildup of ROS and the release of mtDNA into the cytosol. The released mtDNA then triggers the DNA sensor cGAS-STING pathway, which subsequently promotes the production of inflammatory cytokines [16]. The resulting inflammation further inhibits SIRT1, creating a vicious cycle, and drives cells into irreversible senescence. This means that the reduction of SIRT1 does not function independently. It actually triggers a feed-forwarding kind of mechanism that constantly pushes cells into the senescent condition. The tissue environment adds another dimension of complexity to this interaction. In cells that are actively proliferating, a high rate of metabolism leads to an increase in mitochondrial damage and the activation of inflammatory responses. In post-mitotic tissues like neurons, this process occurs at a slower pace. However, the outcomes may be more severe because post-mitotic cells cannot regenerate or replace lost function [17]. Thus, understanding these context-dependent differences is important for developing tissue-specific therapeutic strategies.

This review consolidates the existing knowledge regarding SIRT1’s involvement in cellular senescence, particularly its association with mitochondrial dysfunction and inflammatory signaling. We commence by describing the biology of SIRT1 and the processes that contribute to its age-related reduction. Following this, we focus on three mitochondrial pathways controlled by SIRT1. These are NAD^+^/NADH redox balance, FOXO-dependent antioxidant defense, and PGC-1α-mediated biogenesis, through which SIRT1 exerts its anti-senescent effect. In conclusion, we explore how the reduction of SIRT1 amplifies inflammatory signaling via mtDNA release and the stimulation of the cGAS-STING pathway, while also considering the potential therapeutic implications.

## 2. SIRT1’s Role in Cellular Senescence

### 2.1. SIRT1 Overview and Its Expression

SIRT1 is the mammalian analog of yeast Sir2. It is an NAD^+^-dependent deacetylase and has become an important focus of study in aging. Sirtuins are an evolutionary conserved family of cellular protectors. SIRT1 has various functions within the cell. It is responsible for the deacetylation of histones, transcription factors, and proteins involved in DNA repair. Consequently, it affects energy metabolism, inflammation, and the regulation of the cell cycle [18]. SIRT1’s dependence on NAD^+^ has an important function, making SIRT1 sensitive to metabolism. It can make pivotal decisions on whether a stressed cell should initiate repair processes or proceed towards an irreversible state of cell-cycle arrest [19,20]. This is a central function that explains, in part, the importance of dysregulation of SIRT1, being one of the hallmarks of senescent cells across different tissues of different species.

Senescent cells consistently show diminished levels of SIRT1. Increasing SIRT1 activity can reverse signs of senescence, inflammatory signaling, oxidative stress, and mitochondrial dysfunction. For example, in primary human fibroblasts, SIRT1 levels decrease during both replicative and stress-induced senescence, but its overexpression is able to alleviate the senescent program [10]. This relationship can be observed in vivo: levels of SIRT1 decrease in human peripheral blood mononuclear cells as people age. This decline correlates with p16 expression and telomere shortening [21]. Consistent with these findings, the levels and activity of the SIRT1 protein are diminished in conditions related to aging and oxidative stress, driving cellular senescence [22,23]. Senescent fibroblasts exhibit reduced levels of SIRT1, which demonstrate elevated concentrations of SASP factors [9]. This pattern is not limited to fibroblasts. It appears in stem cell populations. In mesenchymal stem cells (MSCs) from aged rats, both SIRT1 protein and mRNA are reduced. SIRT1 activity dropped by 50% when compared to young cells [23]. Similarly, human MSCs are marked by a reduction in SIRT1 expression as the passage number increases. This reduction is inversely correlated with the levels of p16, p21, and p53 [24]. SIRT1 dysregulation is noted in premature aging syndromes such as Werner syndrome [25] and senescence-accelerated mouse prone (SAMP) models [26], where its decline contributes to accelerated senescence phenotypes.

The relationship between the decrease in SIRT1 and senescence is well-documented. However, correlation does not imply causation. Loss-of-function studies have addressed this question. They show that SIRT1 actively inhibits senescence characteristics. For instance, the silencing of SIRT1 in lens epithelial cells had clear effects. When these cells were exposed to UVB, SIRT1 knockdown increased p53, p21, ROS, and senescence-associated β-galactosidase (SA-β-gal) activity. Even without UVB, the knockdown itself could promote ROS production and cellular senescence [27]. In contrast, the overexpression of SIRT1 in this system led to a decrease in p53 and p21 levels, thereby delaying UVB-induced senescence [27]. There is similar causal evidence in human neural stem cells. siRNA knockdown of SIRT1 increased p21 levels, resulting in an increased percentage of SA-β-gal-positive foci and suggesting a causal relationship [28]. Likewise, in nucleus pulposus cells, the activation of Sirt1 by resveratrol resulted in a decrease in p16 and p21 levels, enhanced cell proliferation, and a reduction in apoptosis [29]. Collectively, these findings establish SIRT1 as a critical gatekeeper of the senescent state. Given that the reduction of SIRT1 greatly accelerates senescence, it is essential to investigate the factors that lead to the initial decrease of SIRT1.

### 2.2. Mechanisms of SIRT1 Decline

The decrease in SIRT1 is influenced by multiple components, including transcriptional repression, microRNA-mediated silencing, post-translational modifications, and depletion of NAD^+^ mechanisms.

#### 2.2.1. Transcriptional Regulation

At the transcriptional level, the circadian clock regulates NAMPT expression. NAMPT is the rate-limiting enzyme in the NAD^+^ salvage pathway, via CLOCK-BMAL1 heterodimers [30]. However, with aging, circadian rhythms become disrupted. This leads to reduced NAD^+^ oscillations and lower levels of SIRT1 activation [31]. Beyond this age-related decline, stress can also affect SIRT1 transcription. Ischemic or oxidative stress can reduce SIRT1 transcription. This happens through SUV39H/HP1γ-mediated trimethylation of H3K9 at the SIRT1 promoter [32]. Transcriptional regulation represents merely one aspect of the overall process. Once SIRT1 mRNA is synthesized, its translation and stability are subject to rigorous control.

#### 2.2.2. Post-Transcriptional Regulation

MicroRNAs play an important role in post-transcriptional silencing. One example is miR-34a. This microRNA binds to the 3′untranslated region (3′UTR) of SIRT1 mRNA and consequently inhibits its expression in models associated with oxidative stress and aging [33]. In the context of neurodegenerative diseases, inflammatory signaling can suppress the activity of SIRT1. This suppression is attributed to the influence of miR-34a and a decrease in the production of NAD^+^. In addition to miR-34a, miR-132 has also been pointed out as another SIRT1 inhibitor [34]. Interference by microRNAs suggests that post-transcriptional repression is not simply a passive trait but a deliberate regulatory mechanism that cells deploy to meticulously check SIRT1 levels in response to stress.

#### 2.2.3. Post-Translational Modifications

Multiple post-translational modifications can influence the stability and the activity of SIRT1. It has been shown that under stress-induced premature senescence, SIRT1 is marked for ubiquitination by a cellular complex called the APC/C-Cdh1 complex and, as a result, is degraded through the proteasome. A nuclear protein called AROS was found to compete with Cdh1, thus blocking the degradation process [11]. Direct alterations that decrease SIRT1 catalytic activity include S-glutathionylation [35], S-nitrosylation [36], and carbonylation [37]. Contrary to this, other alterations promote or stabilize SIRT1, including SUMOylation and stress-induced O-GlcNAcylation [38,39]. All of these indicate that the decline in SIRT1 function is determined by the balance of opposing protein modifications. Crosstalk among these three levels of regulation, providing a fine-tuned system of control over SIRT1 activity, also creates a number of possible points of manipulation that may be taken advantage of throughout the aging process.

#### 2.2.4. Tissue-Specific Consequences and Sirtuin Members’ Adaptive Response

Effects of SIRT1 deficiency vary considerably depending on the tissue environment, highlighting the importance of understanding tissue-specific regulatory mechanisms. For instance, in the aged hippocampus, both SIRT1 and BDNF levels subside, driving impairment of synaptic plasticity and neuronal excitability upon surgery [40]. In the atrial tissue, on the other hand, the loss of SIRT1 promotes RIPK1 acetylation, leading to necroptosis and consequently promoting the development of age-related atrial fibrillation [41]. In the decidualizing endometrium, SIRT1 is downregulated naturally in conjunction with increased levels of FOXO1, highlighting the physiological relevance of SIRT1 downregulation [42]. Synthesizing these pieces of evidence, sustaining optimal levels of SIRT1 expression is critical for healthy aging, although these requirements are dependent on tissue type and situational context. The metabolic demands that are specific to tissues have a considerable impact on the consequences of SIRT1 dysfunction. High-energy tissue like the brain is highly dependent on mitochondria, both in terms of quality control and number, and are therefore particularly sensitive to loss of SIRT1-mediated mitochondrial regulation.

Compensatory mechanisms involving other sirtuin family members can partially compensate for the loss of SIRT1 in certain tissues. This phenomenon has been particularly well-elucidated in heart tissue, demonstrating how SIRT1 and SIRT3 form a dynamic adaptive network in response to ischemia and reperfusion stress. In aged hearts, SIRT1 relocates to mitochondria and recruits more mitochondrial SIRT3 in order to increase their interaction during acute ischemia, allowing for adaptive protection against further mitochondrial dysfunction in aging hearts [43]. This revealed that tissue sensitivity to loss of SIRT1 depends on the differential expression and interactions of sirtuin family members. SIRT3 rests in the mitochondria and is a direct executor of mitochondrial quality control. Mitochondrial SIRT3 controls acetylation of enzymes in oxidative phosphorylation, the TCA cycle, fatty acid oxidation, and antioxidant defense [44,45,46]. SIRT6 operates as a nuclear partner, increases SIRT3 transcription through Nrf2 and AMPK–FOXO3 signaling, and therefore indirectly affects mitophagy [15,47]. Therefore, tissue sensitivity to loss of SIRT1 can depend on a combination of metabolic demand, baseline SIRT1 expression, and functional interactions among sirtuin family members.

Moreover, sex differences add another layer of complexity to tissue-specific SIRT1 sensitivity. SIRT1-mediated protection from age-associated diseases may differ between female and male tissues. For instance, estradiol signaling increases SIRT1 expression by fusing estrogen receptors in various tissues (especially in breast cancer) [48]. This variability in tissue expression, combined with sex-specific differences in the regulation of SIRT1, has implications for considering SIRT1 as a biomarker for aging.

## 3. Mitochondrial Dysfunction and SIRT1

SIRT1 has emerged as a crucial link between metabolism and aging. An NAD^+^-dependent deacetylase, SIRT1 senses the energy status of the cell and transmits that signal to impact transcriptional decisions. One key area for SIRT1-mediated signaling is the activation of PGC-1α. PGC-1α is the main regulator of mitochondrial biogenesis. Furthermore, SIRT1 plays a role in the modulation of PPARα and liver-X-receptors (LXR), which are essential for the regulation of lipid and glucose homeostasis [7]. However, the reduction in NAD^+^ levels associated with aging leads to a decrease in SIRT1 activity, which in turn impairs the activation of PGC-1α and results in lower mitochondrial content. This results in the metabolic rigidity often seen during senescence. The clear contribution of SIRT1 to mitochondrial health is well-documented and involves mechanistic considerations. Below, we explore the contributions of SIRT1 to mitochondrial health as it relates to its impact on (1) NAD^+^/NADH redox balance; (2) FOXO-dependent antioxidant defense and biogenesis; (3) PGC-1α signaling; (4) the process of mitophagy; and (5) how SIRT1 modulates the immune responses associated with mitochondrial dysfunction.

### 3.1. SIRT1 and the NAD^+^/NADH Redox Balance

SIRT1 is an NAD^+^-dependent deacetylase. So, its activity is closely linked to cellular NAD^+^ levels. A decline in NAD^+^ concentrations in redox balance leads to a decrease in SIRT1 activity. This promotes cells to activate pathways associated with p53, p21, NF-κB, and mitochondrial dysfunction that facilitate senescence [14]. This relationship has been observed in several experimental systems. Each system shows a different way that NAD^+^ is depleted. In fibroblasts deficient in NLRX1, failure to maintain the NAD^+^/NADH ratio results in lowered SIRT1 levels and the activation of mTOR, p16, and p53 [49]. In senescent MSCs, miR-34a downregulates NAMPT mRNA, reducing NAD^+^ levels, the NAD^+^/NADH ratio, and SIRT1 activity [50]. Cadmium exposure in fibroblasts causes a decrease in NAD^+^ levels, which in turn reduces SIRT1 levels. This reduction leads to hyperacetylation of p53 and SOD2, impairing mitochondrial function and inducing a state of senescence [51]. Evidence supports the following model. In this context, the presence of sufficient NAD^+^ is essential for the SIRT1-mediated functions related to mitochondria, antioxidant defenses, and anti-senescence signaling pathways. A decrease in NAD^+^ or the onset of redox stress can render cells vulnerable to entering a senescence program [52].

Modulation of NAD^+^ levels may extend beyond increased SIRT1 activation. While nicotinamide can raise NAD^+^ levels in certain stem cell models, it may also feedback to inhibit SIRT1 enzymically, complicating the expected benefits of NAD^+^ repletion. In addition, the benefits seen in MSCs are linked to SIRT3. Therefore, the effects of NAD^+^ precursors are not solely dependent on SIRT1 [52,53]. Despite these complications, NAD^+^ depletion generally raises SASP signaling as reduced SIRT1 activity releases the repression of NF-κB/p65, p53, and other prosenescent transcriptional pathways. Senescence models also show that SIRT1 instability affects IL-6 and IL-8 regulation. This links SIRT1 decline to canonical SASP cytokine production [11,54]. The connection between NAD^+^ depletion and inflammation suggests a broad mechanism of age-related immune dysregulation. There is strong evidence that CD38 upregulation is the main source for NADase activity in aging. In mice, the levels and activity of CD38 protein increase with age in many tissues. PARP1 and SIRT1 seem to depend on this context but are not universally elevated in all tissues [55]. The level of NAD^+^ in mouse embryonic fibroblasts derived from wild-type, CD38, PARP1, and SIRT1 knockout exhibits different levels. Notably, only the CD38 knockout exhibited a significant rise in cellular NAD^+^ levels in comparison to their wild-type control cells. This suggests that a universal model by which NAD^+^ loss is not solely attributed to PARP1 and SIRT1 [55]. Aging leads to the differentiation of immune cells into a CD38-high phenotype, which helps prevent the accumulation of M1-like macrophages in white adipose tissue and the liver. These macrophages exhibit elevated levels of CD38 and demonstrate increased NADase activity [56]. SASP cytokines secreted by senescent cells further enhance this process by promoting the proliferation of macrophages and the expression of CD38 [56,57]. CD38 worsens the depletion of NAD^+^ and NMN, greatly restricting the salvage pathway and resulting in a decrease in the NAD^+^ pool [55,58]. This is not specific to adipose tissue and liver. It also occurs in the ovaries and endometrium. In these tissues, age-related inflammation increases CD38 expression and lowers NAD level [59,60]. However, the exact mechanisms of how the ectoenzyme CD38 on immune cells consumes NAD^+^ remain largely unknown. The NAD^+^-SIRT1 pathway does not work independently. When NAD^+^ levels drop and SIRT1 ceases, cells lose the metabolic flexibility. They also lose the ability to start antioxidant defense program.

### 3.2. FOXO-Dependent Antioxidant Defense

**Figure 1:**
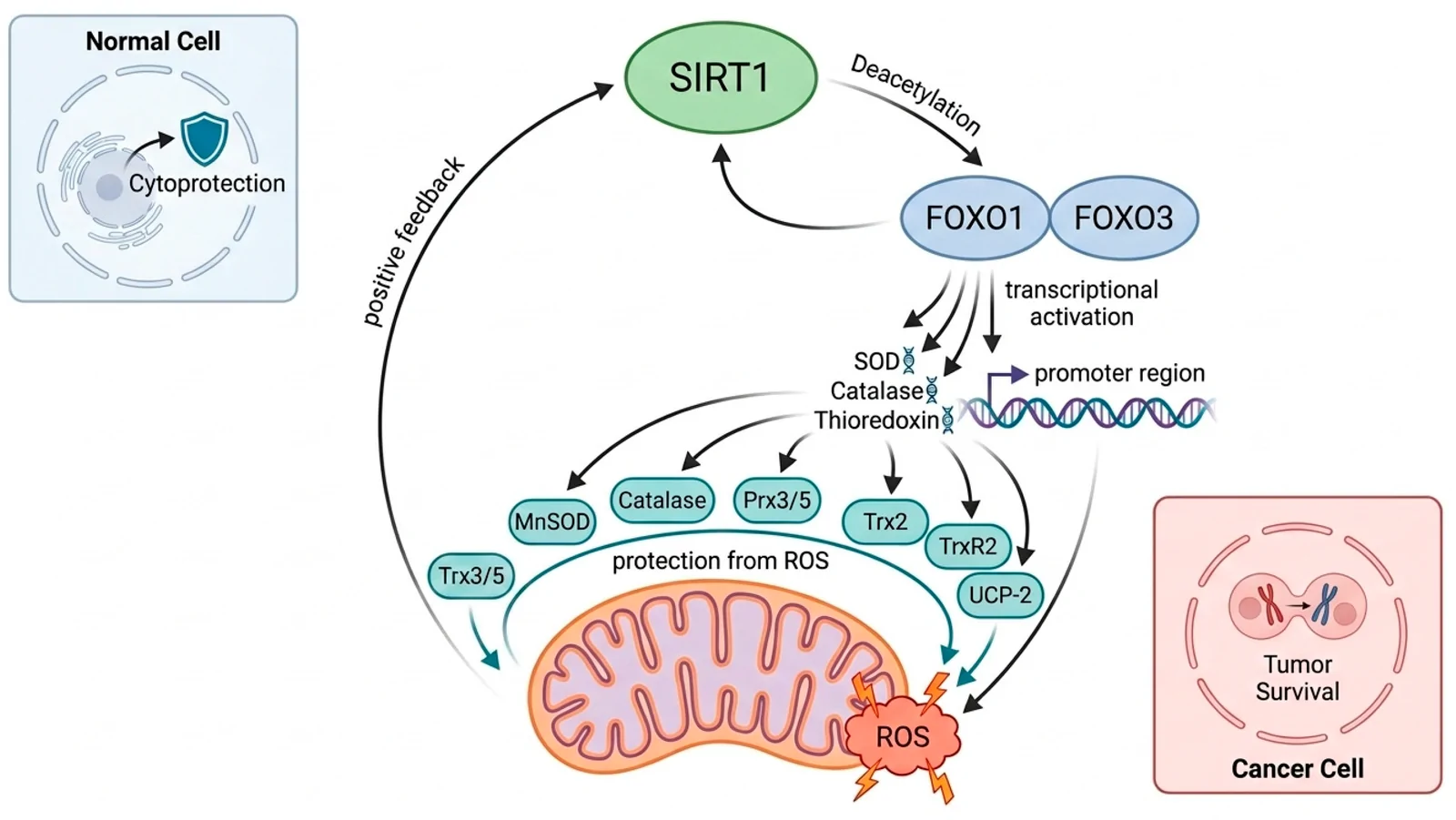
The SIRT1-FOXO antioxidant defense pathway. SIRT1 (green) deacetylates FOXO1 and FOXO3 (blue), activating antioxidant genes (SOD, catalase, thioredoxin) and producing enzymes (MnSOD, Prx3/5, Trx2, TrxR2, UCP-2) that neutralize ROS. In normal cells, this provides cytoprotection. In cancer cells, it suppresses apoptosis while sustaining antioxidant defenses, promoting tumor survival and therapy resistance.

The SIRT1–FOXO signaling pathway represents a persistent antioxidant defense mechanism, as illustrated in Figure 1. Mechanistic evidence is derived from cellular and animal studies. Research demonstrates that SIRT1 activates FOXO transcription factors, increases the levels of antioxidant enzymes like catalase and SOD2/MnSOD, and ultimately attenuates oxidative damage [61,62]. SIRT1 increases the transcriptional activity of the FOXO family of proteins by deacetylating FOXO1 and FOXO3, promoting the expression of genes that favor resistance to oxidative stress [63,64]. FOXOs serve as direct transcriptional regulators of antioxidant programs. This program includes SOD, catalase, and those related to thioredoxin [61,65]. In endothelial cells, SIRT1 modulates a wide range of mitochondrial antioxidant components, including MnSOD, catalase, Prx3, Prx5, Trx2, TrxR2, and UCP-2 [62]. This program relies on a complex FOXO3a and PGC-1α. Silencing either FOXO3a or PGC-1α inhibits the induction of antioxidant genes driven by SIRT1. Furthermore, SIRT1 promotes the formation of this complex while simultaneously decreasing the oxidative stress-dependent acetylation of both components [62]. This regulatory mechanism is strengthened by a positive feedback loop. FOXO1 directly promotes SIRT1 expression. At the same time, SIRT1 deacetylates FOXO1. This boosts FOXO-mediated autoregulation of SIRT1 in vascular smooth muscle cells [66].

The generation of antioxidants is context-dependent. This complexity is well described in cancer models. In cancer models, SIRT1-mediated deacetylation of FOXO1 and FOXO3 can sustain antioxidant defenses while simultaneously suppressing apoptosis or senescence, allowing tumor survival instead of protecting tissues [14]. In acute myeloid leukemia, one study discovered an antioxidant mechanism associated with SIRT1 that is independent of the induction of FOXO antioxidant genes. In this case, FOXO1 relates to SIRT1 without changing the levels of SOD1, SOD2, or catalase, suggesting that SIRT1 antioxidant effects are not always demonstrated through the canonical FOXO transcriptional program [67]. Aside from these exceptions, the general mechanism in most systems is that SIRT1 keeps FOXO factors in a deacetylated and transcriptionally active state, leading to the expression of antioxidant genes, supporting its action in restraining ROS buildup [14,63].

### 3.3. The PGC-1α Pathway: Important, but Not Obligatory in Some Contexts

The effect of SIRT1 deficiency on mitochondria is paramount. The generally accepted model states that SIRT1 deacetylates PGC-1α (the main orchestrator of mitochondrial biogenesis), resulting in the initiation of a transcriptional program including NRF1, TFAM, and PPARα, all of which contribute to the generation and maintenance of the mitochondrial network [13,68,69]. For instance, in pancreatic cells, resveratrol enhanced the activity of SIRT1, decreased the acetylation of PGC-1α and increased downstream targets; these advances were lost when SIRT1 was blocked [70]. Similarly, mechanical stimulation through Piezo1 increased the function of SIRT1 and decreased the acetylation of PGC-1α in bone marrow stromal cells; such effects were attenuated when SIRT1 was inhibited [71]. In a model of a prion disease, SIRT1 protein and activity were shown to diminish, whereas enhancing SIRT1 level can restore mitochondrial architecture and functions affected by the neurotoxic peptide PrP (106-126) [72]. When SIRT1 fails to master mitochondrial homeostasis protective machinery, dysfunctional mitochondria thus begin to accumulate, ROS production increases [73], and mitochondrial DNA leaks out to cytosol.

A genetic study conducted on skeletal muscle following endurance training revealed that SIRT1 surprisingly was non-essential. PGC-1α was able to continue undergoing deacetylation, and mitochondria were able to continue multiplying, likely as a result of reduced GCN5 activity [74]. Even in liver cells, SIRT1 was unable to alter the acetylation of PGC-1α in a context of TRβ1 coactivation, implying that some of the transcriptional actions of SIRT1 occur alongside and not via PGC-1α [75]. Notwithstanding, the vast majority of studies on adipocytes, hypoxic heart tissue, pulmonary vascular cells, and circadian neurons consistently assert that SIRT1 is situated firmly upstream of PGC-1α-dependent mechanisms [76,77,78,79]. An apparent paradox arises from findings that increased PGC-1α expression can be found in some senescent cells even when SIRT1 activity is reduced. PGC-1α expression increases in certain metabolically stressed or senescent cells even when SIRT1 activity declines, decoupling these two factors from their canonical positive-feedback loop [80,81]. These examples illustrate that increased PGC-1α in the context of low SIRT1 reflects a dysregulated stress response rather than productive mitochondrial renewal. Therefore, functional quality of newly generated mitochondria determines whether the outcome is adaptive or pathological.

### 3.4. Mitophagy: When Quality Control Fails

**Figure 2:**
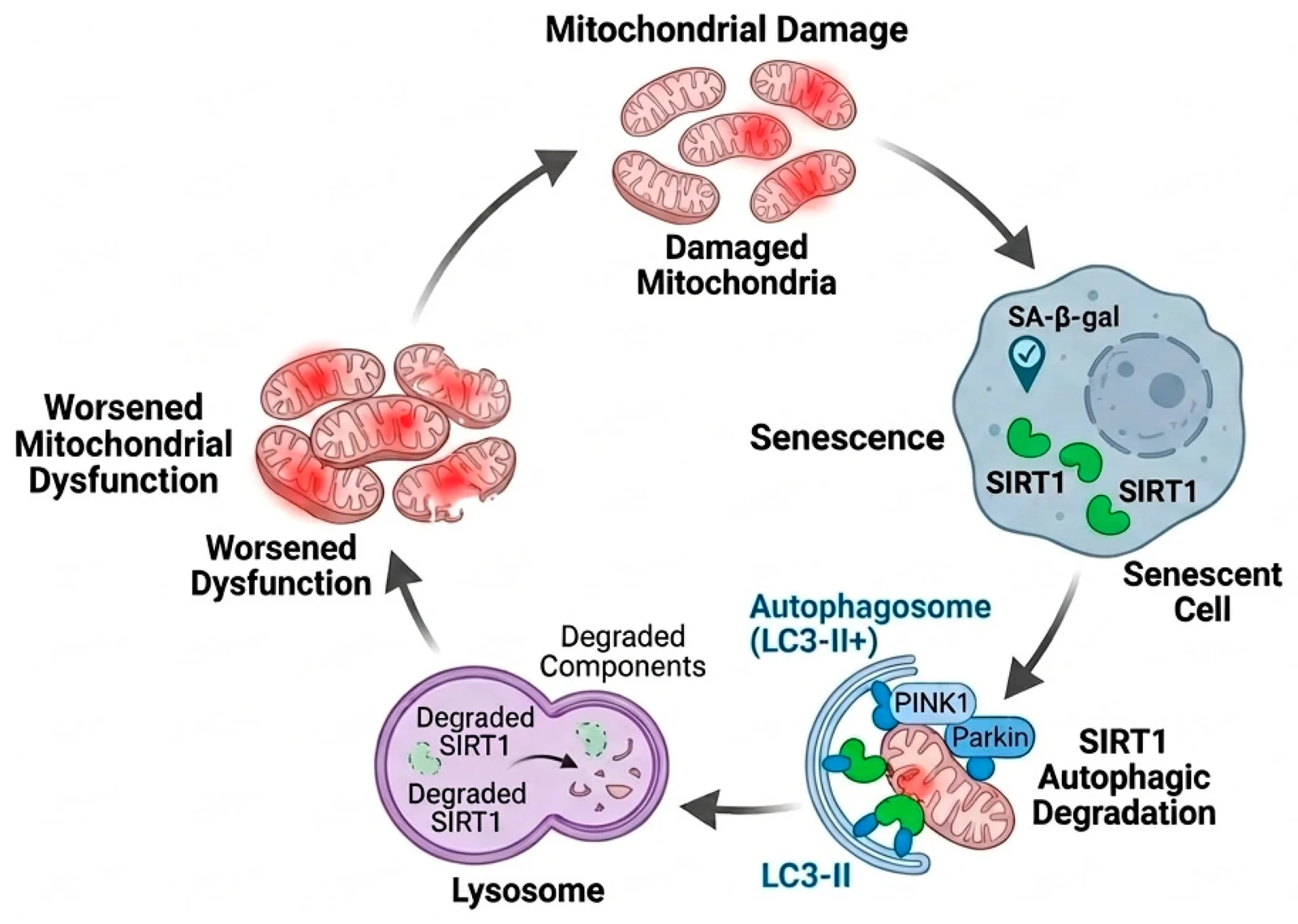
The SIRT1–mitophagy–senescence feedback loop. SIRT1 (green) promotes mitophagy via PINK1/Parkin and LC3-II (blue), maintaining mitochondrial quality. SIRT1 loss leads to damaged mitochondria (red) and senescence (indicated by SA-β-gal positive cell, blue check mark). Senescent cells degrade SIRT1 through autophagy: nuclear SIRT1 binds LC3 (blue), exits the nucleus, and enters the lysosome (purple). This creates a self-reinforcing loop.

In addition to boosting mitochondrial biogenesis, SIRT1 is also proven experimentally to promote the elimination of damaged mitochondria via a mechanism called mitophagy, as illustrated in Figure 2. The evidence strongly supports this claim [27,82,83], albeit still lacking human trial data and originating mostly from cell culture and animal studies. When SIRT1 is either silenced or inhibited, this leads to a buildup of damaged mitochondria, increased ROS, and inflammation markers, all factors known to induce cell senescence. In auditory cells, the activation of SIRT1 not only protects against senescence but simultaneously induces an increase in mitophagic markers such as PINK1, Parkin, BNIP3, and LC3-II. Silencing SIRT1 can block this protective effect, suggesting SIRT1-dependent mitophagy [82]. A similar finding was also noted in nucleus pulposus stem cells, where the silencing of SIRT1 had a deleterious effect on Parkin-mediated mitophagy, contributing to pronounced oxidative senescence in the cells [84]. Likewise, in vascular endothelium, estrogen anti-aging functions were mediated via a SIRT1/LKB1/AMPK/Ulk1 mitophagy pathway [83].

Impaired mitophagy and senescence are related to each other even in the absence of SIRT1 manipulation. Senescent and naturally aged primary human cells display decreased steady-state levels of PINK1/Parkin-p62 mitophagy. Indeed, direct disruption of mitophagy even in healthy and dividing cells can initiate the senescence program [82]. This is an important finding, highlighting the fact that mitophagy is not a mere downstream consequence but a central gatekeeper to the senescent state. However, the relationship between SIRT1 and mitophagy is not one way. Senescent cells are not only characterized by loss of SIRT1 activity, but cells also enhance degradation of the protein through autophagy (Figure 2). SIRT1 in the nucleus interacts with LC3, leaves the nucleus, and heads to the lysosome for destruction [10,85]. This is not just a cell culture artifact, as a declination is seen in the spleen and thymus of aged mice, as well as in aged hematopoietic stem and human CD8^+^CD28^−^ T cells [85]. Inhibition of autophagy via ATG7 depletion prevents SIRT1 loss, while mutations of the LC3 interacting region cause SIRT1 to remain in the nucleus [10]. The autophagic degradation of SIRT1 is not simply a passive association with senescence; rather, it plays an active role in fostering a detrimental feedback loop. Consequently, a detrimental feedback loop is established where mitochondrial impairment induces senescence. Senescence facilitates the autophagic degradation of SIRT1, and the loss of SIRT1 further aggravates mitochondrial dysfunction. This cycle is demonstrated in alveolar epithelial cells subjected to cigarette smoke stress, where diminished mitophagy leads to elevated mtROS, DNA damage results in decreased NAD^+^/NADH levels, and SIRT1 activity declines, perpetuating a feedback loop with autophagy [86]. The claim that senescence results in the autophagic breakdown of SIRT1 is most compellingly shown in this model, and it appears to be context-dependent rather than a universal principle that applies to all senescent cells. Promisingly, the pharmacological stimulation of SIRT1-mediated autophagy using melatonin, metformin, or rhapontigenin reliably diminishes oxidative stress via reducing levels of p53/p21 and markers of senescence [87,88,89]. SIRT1 plays a crucial role in preserving mitochondrial integrity via mitophagy, and any disturbance in this process can lead to cellular senescence; furthermore, the consequences of impaired mitophagy extend beyond the mitochondria.

### 3.5. SIRT1 and Immune Activation in Cellular Senescence

SIRT1 deficiency impairs mitochondrial quality control and induces the development of cellular senescence through a self-maintained feedback loop. This feedback loop integrates the mitochondrial pathways with inflammatory signaling, creating a cycle that perpetuates the state of senescence.

#### 3.5.1. The mtDNA-cGAS-STING Axis and SASP Induction

**Figure 3:**
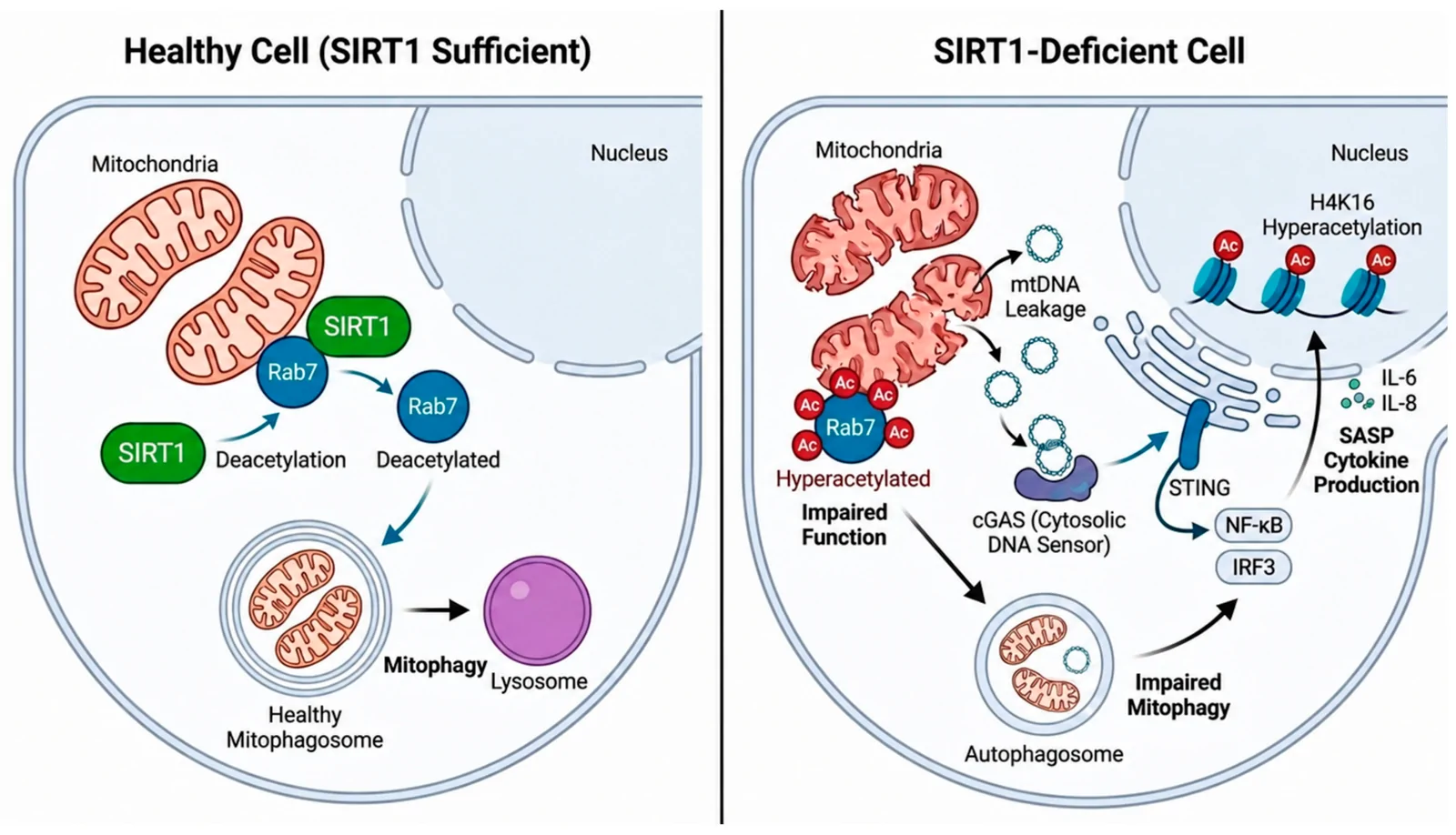
The mtDNA-cGAS-STING axis in SIRT1 deficiency. Left panel: Healthy cell with sufficient SIRT1. In healthy cells, SIRT1 promotes mitophagy via Rab7 deacetylation, preventing mtDNA release. Right panel: SIRT1-deficient cell. When SIRT1 is lost, Rab7 becomes hyperacetylated, impairing mitophagy, allowing mtDNA to activate cGAS-STING, leading to NF-κB/IRF3 nuclear translocation and SASP cytokine production (IL-6 and IL-8). SIRT1 also maintains repressive chromatin via H4K16 deacetylation; its loss opens chromatin for NF-κB binding.

The cascade begins with the loss of SIRT1, which causes mitochondrial breakdown. Leaked mtDNA serves as a danger signal: its unmethylated CpG motifs stimulate TLR9, whereas double-stranded mtDNA activates the cGAS-STING pathway, resulting in the release of type I interferons (IFNs) and pro-inflammatory cytokines [90]. The cGAS-STING pathway has been identified as an important bridge between mitochondrial dysfunction and SASP [91]. The deficiency of SIRT1 may affect cGAS-STING pathway in three different ways. It elevates the levels of mtROS, which in turn harms mtDNA; it inhibits mitophagy, allowing the presence of damaged mitochondria accumulation; and it leads to the loss of deacetylation capacity of STING, leading to the hyperactivity of the pathway, as illustrated in Figure 3 [92]. In lung endothelial cells, a lack of SIRT1 aggravates mitochondrial injury, increases mtROS, and causes the release of cytosolic mtDNA, which leads to the excessive activation of both STING and NLRP3 inflammasomes [16]. Mechanistically, SIRT1 initiates the deacetylation of Rab7 to promote mitophagy. In the absence of SIRT1, acetylation of Rab7 hinders the process of mitophagic clearance, resulting in the accumulation of damaged mitochondria and leakage of mtDNA into the cytosol [16]. Consequently, cytosolic mtDNA engages cGAS, generating 2′3′-cGAMP that activates STING. Subsequently, STING recruits TBK1 and IKK, leading to the nuclear translocation of IRF3 and NF-κB and the transcription of type I interferons and pro-inflammatory cytokines (IL-6, IL-8, IL-1β)—the core SASP components [91]. Similarly, causal evidence comes from ocular inflammation models substantiates that mtDNA is essential and sufficient for STING-dependent inflammation [93].

SIRT1 also mediates epigenetic repression of SASP. In homeostasis, SIRT1 deacetylates H4K16 at the SASP promoter, thereby retaining repressive chromatin for NF-κB binding, as illustrated in Figure 3. When SIRT1 is lost, histones become hyperacetylated and allow for the recruitment of NF-κB, thereby rapidly switching on SASP genes [9]. This epigenetic alteration often acts as a threshold switch. Downregulation of SIRT1 is frequently detected in senescent cells, which tend to act as a signal to the immune system to alert it for destruction, but persistent accumulation can increase inflammation [10]. This can be seen consistently across multiple models. Lens epithelial cells exposed to UVB, cadmium-treated fibroblasts, adipogenic MSCs, and Aβ-stimulated microglia all have reduced levels of SIRT1 alongside oxidative stress and senescence mediated by p53/p21/p16 [27,51,94,95].

#### 3.5.2. Immune-Mediated Suppression of SIRT1 and Its Consequences for Immune Cell Function

The self-amplifying nature of the SIRT1-inflammation loop is further reinforced by NF-κB; gain-of-function studies show that overexpression of SIRT1 interrupts this cycle, leading to a decrease in ROS, which aids in maintaining mitochondrial membrane potential and improves PINK1-Parkin mitophagy [96]. This mitigates senescence in nucleus pulposus cells subjected to stress and fibroblasts exposed to cadmium [51,97]. In models of immune responses, stroma, and aging, inflammatory signal triggers a reduction in SIRT1 level through multiple mechanisms, including inhibiting its expression, reducing its NAD^+^-dependent activity, or accelerating its degradation, which in all turn result in the activation of NF-κB, inflammasome, and SASP activation pathways [22,98].

Not limited to stroma cells, this SIRT1 decline also affects directly the function and senescence of immune cells. In macrophages, SIRT1 activation favors macrophage polarization towards the anti-inflammatory M2 phenotype and inhibits pro-inflammatory M1 activation [99]. In CD4+ T cells from Hepatitis C virus-infected patients, SIRT1 is upregulated in a paradoxical counterregulatory way in response to accelerated senescence, therefore silencing SIRT1 increases T cell senescence [100]. In NK cells, SIRT1 expression is significantly increased in aged people compared to younger age groups, suggesting that SIRT1 may play a role on cellular homeostasis biology. This situation is confirmed by the positive correlation of SIRT1 expression and age, which is generally accompanied by elevated expression of other protective cellular proteins including HSP70 and SOD2 [101].

The most well-supported direct turnover pathways include LC3-dependent autophagic degradation in senescent and aging tissues, particularly within immune compartments, as well as APC/C-Cdh1-dependent proteasomal degradation, which occurs during stress-induced senescence [10,11]. Upstream immune signals can intervene beyond protein degradation, either by regulating protein expression or post-transcriptionally modifying its mRNA. In an ischemia-stressed liver model, Ikaros factor in recruited macrophages drove pyroptosis and liver inflammasome activation in vivo via AMPK-dependent SIRT1 suppression [102]. Senescent cell-derived extracellular vesicles induced IL-1β/IL-6 rise and SIRT1 decline but were reversed by SIRT1 activation or miR-30b-5p blockade [102,103]. These studies demonstrate that inflammatory signals actively suppress SIRT1 activation through multiple connected mechanisms, reinforcing the self-amplifying cycle driving senescence. Conversely, SIRT1 loss in immune cells drives a pro-inflammatory phenotype and accelerates immune senescence, creating a bidirectional loop linking systemic inflammation to immune dysfunction.

## 4. Therapeutic Implications

### 4.1. Natural SIRT1 Activators

The direct activation of SIRT1 has been explored through two primary approaches: the use of natural phytochemicals and synthetic sirtuin-activating compounds (STACs). Natural products, particularly polyphenols, have attracted a lot of attention, but most of the evidence remains at a preclinical stage, and it is thus not possible to isolate the precise role of SIRT1 itself [104,105,106]. This review has mostly focused on the natural SIRT1 activators.

Among the natural compounds reported to activate or upregulate SIRT1 in the context of metabolic and neurodegenerative diseases, resveratrol is the best-known example [104,105,106]. Natural SIRT1 activators are summarized in Table 1. Resveratrol is often referred to as an allosteric SIRT1 activator, but because of its poor pharmacokinetics and pleiotropic biology, it is difficult to specifically attribute benefits to direct SIRT1 activation [107,108,109]. This raises critical questions regarding the physiological relevance of direct SIRT1 activation and suggests that other mechanisms, such as AMPK activation, may be involved. In models of disease, direct activation of AMPK was shown to restore Nrf2 levels in frataxin-deficient neurons with a specific AMPK activator, supporting the AMPK-Nrf2 coupling, although the SIRT1 was reduced [110]. Resveratrol has been shown to activate AMPK independently of SIRT1. Studies conducted with primary human myotubes showed that resveratrol promoted AMPK phosphorylation in both lean and severely obese women, whereas SIRT1 levels increased only in lean cells. This suggests that AMPK activation can occur without SIRT1 upregulation simultaneously in obesity. This finding presents challenges to the specific correlation of effects with SIRT1 [111].

Similar uncertainties also surround the actions of other polyphenols, like quercetin, which has been linked to benefits in human endothelial and liver cell lines in in vitro studies, but the related mechanism with SIRT1 is not fully elucidated [112,113]. Naringenin has been documented to activate SIRT1 in the human cardiomyocyte cell line and confer protective effects against myocardial dysfunction related to senescence in animal models, with in silico studies corroborating its binding on SIRT1 [114]. Hydroxytyrosol has been associated with activation of SIRT1 in several models, including human primary chondrocyte, human endothelial cell, human and rat cartilage cell, and AGE-toxicity models in human neuroblastoma cell line and animal models; it is worth noticing, however, that the evidence reported herein is still preclinical [115,116,117,118].

A striking example of direct binding with SIRT1 originates from Thai black ginger. The active component, quercetin 3,5,7,3′,4′-pentamethyl ether (KPMF-8), binds directly with SIRT1, which leads to an increase in SIRT1 activity toward a native p53 peptide and a rise in intracellular deacetylase activity when compared with resveratrol in the same evaluation [119]. Curcumin has been shown to exert SIRT1-dependent effects in numerous models. In pathological models of myocardial ischemia–reperfusion, curcumin was shown to reduce mitochondrial oxidative damage and maintain mitochondrial redox potential through SIRT1 signaling [120]. In models of cardiac dysfunction by sepsis, curcumin has been shown to activate SIRT1 and subsequently promote mitochondrial biogenesis, decrease the translocation of DRP1 to mitochondria, and improve cardiac function [121]. In models of kidney injury, curcumin has been shown to activate the SIRT1/Nrf2/HO-1 pathway, which counteracts oxidative stress, mitochondrial damage and apoptosis induced by the nephrotoxin aristolochic acid [122]. Studies further found that polymethoxyflavonoids from citrus peel could also activate SIRT1 with heptamethoxyflavone being more effective than resveratrol [123].

A commonly noted drawback of these natural substances is their limited bioavailability. Several effects of polyphenols reported in vitro achieve concentrations normally ranging between 0.25 and 100 µM; however, these concentrations may not be achieved in vivo. This dissimilarity between in vitro efficacy and in vivo levels underlines the need for caution when considering mechanistic studies and highlights the importance of pharmacokinetic factors in translational studies. Most of the evidence comes from in vitro or animal studies. These limitations have spurred the development of more selective synthetic SIRT1 activators. Collectively, these limitations highlight the need for improved bioavailability and more selective ways of targeting SIRT1 including STACs, though the therapeutic potential of natural SIRT1 activators is an active area of investigation.

**Table 1:** Natural SIRT1 activators: mechanisms, effects, evidence level, and concentration.

Compound	Proposed Mechanism	Key Effects	Evidence Level and Concentration
Resveratrol	Activates AMPK, modulates NF-κB [124,125]	Reduced mitochondrial functions, bone formation [124,125]	Murine muscle cell line (25 µM), bone-derived cell (5 µM), animal model (25–30 mg/kg)
Quercetin	SIRT1 and AMPK activation [112,113]	Reduced NF-κB activation; protects against mitochondrial dysfunction, and suppresses NLRP3 inflammasome activation, protecting liver injury [112,113]	Human endothelial cell line (10 µM), human liver cell line (5–10 µM), animal model (100 µg/mL, 50–100 mg/kg)
Naringenin	SIRT1 activation [114]	Reduced ROS production and inflammatory markers; protects against senescence-related myocardial dysfunction [114]	Human cardiomyocyte cell line (40 µM), animal model (100 mg/kg)
Curcumin	SIRT1 activation [120]	Reduced mitochondrial oxidative damage; maintained mitochondrial redox potential [120]	Rat normal isolated heart cell (0.25–1 µM), animal model (220–250 g/kg)
Curcumin	SIRT1 activation; mitochondrial biogenesis promotion [121]	Decreased DRP1 translocation to mitochondria; enhanced cardiac function; promoted mitochondrial biogenesis [121]	Human cardiomyocyte cell line (20 µM), animal model (80 mg/mg)
Curcumin	SIRT1/Nrf2/HO-1 pathway activation [122]	Counteracted oxidative stress; counteracted mitochondrial damage; counteracted apoptosis [122]	Rat kidney cell line (20 µM), animal model 200 mg/kg)
Heptamethoxyflavone (HMF)	SIRT1 activation [123]	Strongest SIRT1 activation among PMFs; surpasses resveratrol [123]	Human hepatocarcinoma cell line (12.5–100 µM)
Hydroxytyrosol	post-transcriptionally regulates SIRT-1 [115]	Decreased miR-9 levels; increased SIRT-1 levels; reduced oxidative stress-induced chondrocyte death [115]	Human primary chondrocyte and human cartilage cell line (10 µM)
Hydroxytyrosol	SIRT1 activation [116]	Decreases blood glucose and oxidative stress in diabetic models [116]	Human endothelial cell line (50 µM), animal model (77 mg/kg)
Hydroxytyrosol	Counteracts AGE-induced cytotoxicity in neurotypical cells by acting on SIRT1 level [117]	Decreased protein glycation (human insulin) and AGE-induced cytotoxicity; reduced inflammatory response; Neuroprotection [117]	Human neuroblastoma cell line (0.5–2 mM)
Hydroxytyrosol	SIRT activation [118]	Increased autophagy and decreased Inflammatory cytokines; chondroprotection [118]	Rat primary cartilage cell (75 µM)
KPMF-8	Direct SIRT1 binding; enhanced binding to p53 peptide 8.2-fold; increased deacetylase activity 1.7-fold [119]	Direct SIRT1 activation; outperforms resveratrol in activity assays [119]	Human breast cancer cell line (20 µM)

Animal Models: In vivo studies using rats or mice to assess physiological or disease-relevant effects.

### 4.2. Safety Considerations and Translational Challenges

Beyond the practical issues associated with pharmacokinetics and clinical translation, there is a more fundamental, biological problem that complicates targeting SIRT1. SIRT1 has a dual role in cancer biology and may function either as a tumor suppressor, by deacetylating p53 and facilitating DNA repair, or as a tumor promoter, through FOXO-mediated antioxidant defense and metabolic reprogramming [14]. This duality creates a therapeutic paradox. Unrestrained activation of SIRT1 may contribute to cancer cell survival by blocking apoptosis and increasing oxidative stress resistance, while inhibition of SIRT1 could make tumors more responsive to treatment but will likely also hasten senescence across healthy tissues. It is probable that establishing the ideal timing, dosage, and tissue specificity for such an intervention will be essential to ensure its safety and efficacy. However, the existing clinical evidence for SIRT1 modulators in humans is limited. In a 28-day Phase I trial of the synthetic SIRT1 activator SRT2104 given to elderly volunteers, the compound was safe and well-tolerated and was associated with reductions in serum cholesterol, LDL, and triglyceride levels [126].

Further, while SIRT1 features prominently in much of the literature to date, accumulating evidence suggests that other members like SIRT3 also play key, non-redundant roles in controlling mitochondrial quality control, maintenance of genomic stability, and inflammation [52,53]. Thorough understanding of how members of the sirtuin family “work together” and “compete” against one another will be critical to fully appreciate the regulatory networks controlling cellular senescence.

## 5. Future Perspectives

The NAD^+^-SIRT1–mitochondria–immune axis is certainly among the most compelling and well-defined mechanistic pathways linking metabolism to aging. As we have reviewed, SIRT1 decline is both a cause and a consequence of cellular senescence, seen through a set of interlocked feedback loops that converge on amplifying mitochondrial dysfunction, inflammatory signaling, and epigenetic reprogramming. Understanding the precise molecular wiring of this axis, including the threshold-dependent switches, tissue-specific regulators, and temporal dynamics, will be essential for developing interventions to promote healthy aging while avoiding unintended consequences. Future studies should also address sex- and tissue-specific differences in SIRT1 regulation.

In the coming years, we expect to gain further insight into the context-dependent functions of SIRT1, with the advent of single-cell technologies, spatial transcriptomics and tissue-specific genetic models. This will help us understand the function of SIRT1 in particular cell types and distinct stages of senescence, beyond the simplistic descriptions of SIRT1 as protective versus pathogenic. Concurrent efforts are underway to develop additional selective SIRT1 modulators, CD38 inhibitors, and enhancers of mitophagy, either alone or in combination with existing senolytic therapies. These help us to improve these mechanistic understandings in clinical practice in order to extend health span and delay or prevent age-related diseases. The road to achieving such future promises is paved with major challenges but also opportunities for paradigm-shifting therapeutic interventions.

## 6. Conclusions

The evidence presented here identifies SIRT1 as a pivotal element linking metabolic sensing, mitochondrial quality control, and inflammation in cellular senescence. Its decline is not merely a consequence of age but actively propagates accumulation of senescent cells by connecting several intersecting mechanisms of transcriptional repression, post-transcriptional silencing, post-translational degradation, and NAD^+^ depletion. These collectively form a self-reinforcing cycle of mitochondrial dysfunction and inflammatory signaling. Once the cycle is initiated, cells cross a threshold to an irreversible state of senescence, which is characterized by activation of the SASP, failure of mitophagy, and a state of persistently low-grade inflammation. When SIRT1 is in a homeostatic state, mitochondrial quality control and antioxidant defenses are maintained and inflammatory signaling is restrained. However, when SIRT1 is depleted via age-related declines in NAD^+^ or perhaps due to chronic inflammation, the feedback loop is compromised; mitochondrial ROS and cytosolic mtDNA, as well as inflammation, can propagate, leading to a senescent state. This homeostatic behavior explains why even small changes in SIRT1 levels can strongly affect cell fate. It also explains why SIRT1 restoration, even when it occurs late in senescence, can still partially improve the senescent phenotype.

## Data Availability

No new data were created or analyzed in this study. Data sharing is not applicable to this article.

## Abbreviations

The following abbreviations are used in this manuscript:

2′3′-cGAMP	2′,3′-Cyclic Guanosine Monophosphate-Adenosine Monophosphate
AGE	Advanced Glycation End-Products
AMPK	Adenosine Monophosphate-Activated Protein Kinase
APC/C	Anaphase-Promoting Complex/Cyclosome
AROS	Active Regulator of SIRT1
ATG7	Autophagy Related 7
Aβ	Amyloid-Beta
BDNF	Brain-Derived Neurotrophic Factor
BMAL1	Brain and Muscle ARNT-like Protein 1
BNIP3	Bcl-2 Interacting Protein 3
CD38	Cluster of Differentiation 38
Cdh1	Cadherin 1
cGAS	Cyclic GMP-AMP Synthase
CLOCK	Circadian Locomotor Output Cycles Kaput
CpG	Cytosine-phosphate-guanine
DNA	Deoxyribonucleic Acid
DRP1	Dynamin-Related Protein 1
EX-527	Selective SIRT1 Inhibitor
FOXO	Forkhead Box O
FOXO1	Forkhead Box O1
FOXO3	Forkhead Box O3
FOXO3a	Forkhead Box O3a
FOXP1	Forkhead Box protein P1
GCN5	General Control Nonderepressible 5
H3K9	Histone H3 Lysine 9
H4K16	Histone H4 Lysine 16
HO-1	Heme Oxygenase 1
HP1γ	Chromobox Protein Homolog 3
IKK	I Kappa B Kinase
IL-1β	Interleukin-1 Beta
IL-6	Interleukin-6
IL-8	Interleukin-8
IRF3	Interferon Regulatory Factor 3
LC3	Microtubule-associated Protein 1A/1B-light Chain 3
LC3-II	Microtubule-associated Protein 1A/1B-light Chain 3-II
LKB1	Liver Kinase B1
LXR	Liver X Receptor
miR-132	microRNA-132
miR-34a	microRNA-34a
MnSOD	Manganese Superoxide Dismutase
mRNA	Messenger Ribonucleic Acid
mtDNA	Mitochondrial Deoxyribonucleic Acid
mTOR	Mammalian Target of Rapamycin
mtROS	Mitochondrial Reactive Oxygen Species
NAD^+^	Nicotinamide Adenine Dinucleotide
NADase	NAD^+^ Nucleosidase
NAMPT	Nicotinamide Phosphoribosyltransferase
NF-κB	Nuclear Factor Kappa-light-chain-enhancer of Activated B cells
NLRP3	Nucleotide-binding domain, Leucine-rich–containing Family, Pyrin Domain–containing-3
NLRX1	NOD-Like Receptor X1
NMN	Nicotinamide Mononucleotide
NRF1	Nuclear Respiratory Factor 1
Nrf2	Nuclear Factor Erythroid 2-Related Factor 2
O-GlcNAcylation	O-linked β-N-acetylglucosaminylation
p16	Cyclin-dependent Kinase Inhibitor 2A
p21	Cyclin-dependent Kinase Inhibitor 1
p53	Tumor Protein p53
p62	Sequestosome-1 (Autophagy Adaptor)
p65	NF-κB p65 subunit
Parkin	E3 Ubiquitin Ligase
PARP	Poly (ADP-ribose) Polymerase
PGC-1α	Peroxisome Proliferator-Activated Receptor Gamma Coactivator 1-alpha
PINK1	PTEN-induced Putative Kinase 1
PPARα	Peroxisome Proliferator-activated Receptor Alpha
PrP (106-126)	Human Prion Protein Fragment 106–126
Prx3	Peroxiredoxin 3
Prx5	Peroxiredoxin 5
Rab7	A Member of the RAB Family of GTPases
RIPK1	Receptor-interacting Serine/threonine-protein Kinase 1
RNA	Ribonucleic Acid
ROS	Reactive Oxygen Species
S-glutathionylation	Post-translational Modification Involving Addition of Glutathione
S-nitrosylation	Post-translational Modification Involving Addition of Nitric oxide
SA-β-gal	Senescence-Associated β-Galactosidase
SASP	Senescence-associated Secretory Phenotype
siRNA	Small Interfering RNA
SIRT1	Sirtuin 1
SIRT3	Sirtuin 3
SIRT6	Sirtuin 6
SOD1	Superoxide Dismutase 1
SOD2	Superoxide Dismutase 2
STACs	Sirtuin-Activating Compounds
STING	Stimulator of interferon genes
SUMOylation	Post-translational Modification Involving Small Ubiquitin-like Modifier
SUV39H	Suppressor of Variegation 3-9 Homolog
TBK1	TANK-binding kinase 1
TFAM	Mitochondrial Transcription factor A
TLR9	Toll-like Receptor 9
Trx2	Thioredoxin-2
TrxR2	Thioredoxin Reductase 2
TRβ1	Thyroid Hormone Receptor Beta-1
UCP-2	Mitochondrial Uncoupling Protein-2
Ulk1	Unc-51 Like Autophagy Activating Kinase 1
UTR	Untranslated Region
UVB	Ultraviolet B Radiation
